# Outcomes of early-stage oropharyngeal squamous cell carcinoma patients treated with radical radiation - a single institution experience

**DOI:** 10.3332/ecancer.2025.1901

**Published:** 2025-04-25

**Authors:** Zuzaki Shabin, Rejnish Kumar, Malu Rafi, Lekha M Nair, Krishnapriya Pisharody, Nijo Jose, Indu Sasikumar, John Mathew, Kumara Pillai Mohanan Nair, Kainickal Cessal Thommachan

**Affiliations:** 1 Division of Radiation Oncology, Regional Cancer Center, Level 8, Regional Cancer Centre, Medical College Campus Road, Trivandrum 695011, Kerala, India; 2 Division of Cancer Epidemiology and Biostatistics, Regional Cancer Center, Level 8, Regional Cancer Centre, Medical College Campus Road, Trivandrum 695011, Kerala, India

**Keywords:** squamous cell carcinoma, early-stage oropharyngeal cancer, radical radiation, outcomes

## Abstract

**Purpose:**

Treatment options for early-stage oropharyngeal squamous cell carcinomas (OPSCC) (Stages I and II) include definitive radiation or surgery. This retrospective study aims to evaluate the outcomes of early-stage (Stages I and II) OPSCC treated with radical radiation.

**Materials and methods:**

The data of patients with early-stage OPSCC (T1/T2N0) treated with definitive radiation at the Regional Cancer Center, Trivandrum, Kerala, India, from 1st January 2015 to 31st December 2020 were retrieved from medical records. A structured proforma was used to gather clinical and therapeutic details of the patients. The primary objective was to assess loco-regional control and patterns of relapse. Secondary objectives were to assess overall survival (OS), disease-free survival (DFS) and prognostic factors affecting the treatment outcomes. DFS and OS were generated using Kaplan–Meier Curves. The prognostic factors affecting the outcomes were analysed using the Cox-proportional hazards regression model.

**Results:**

One hundred and twelve patients with early-stage oropharyngeal cancers who received definitive radiation were included in the study. The majority (91.97%) were males and 90% (101) of the patients had tobacco and alcohol-related habits. Twenty-six (23.3%) among the 112 patients had stage I disease and 86 (76.7%) had stage II disease. The median age group of the study population was 62 years. The most commonly affected primary site was the tonsil (*N* = 38, 33.9%), followed by the soft palate (*N* = 34, 30.3%) and followed by the base of the tongue (*N* = 23, 20.5%). The majority of the patients (80.4% (*N* = 90)) received a radiotherapy (RT) dose of 60 Gy in 26 fractions at 2.3 Gy per fraction over 5.5 weeks. One hundred nine (97.3%) patients attained remission at 12 weeks following radiation. Three patients had residual disease and none of them underwent salvage surgery. Twenty-one percent (*N* = 24) of patients had relapsed and the median time to relapse was 20 months. Among the relapses, 7 (29%) underwent salvage surgery and others were given palliative treatment. The most frequent site of relapse was the primary site followed by regional nodes. At a median follow-up of 63 months, the 4-year DFS was 67.9% and OS was 75%. Stage-wise 4-year DFS and OS for stages 1 and II were 68.1% and 64.1%, 78.9% and 73.8%, respectively. The locoregional relapse-free survival at 4 years was 75.2%. Five patients developed a second malignancy and the lung was the most common site. In univariate analysis, age was the only significant prognostic factor that affected the treatment outcomes.

**Conclusion:**

The survival outcomes of the patients treated with definitive RT are comparable with the published literature. However, the salvage rates were very poor.

## Background

Head and neck cancers are the seventh most prevalent cancer worldwide and around 10% of them are oropharyngeal squamous cell carcinomas (OPSCC) [1, 2]. Men are more likely than women to develop OPSCC, with peak incidence occurring in the sixth and seventh decades of life [3–6]. Due to the rich lymphatic network in the oropharyngeal region, OPSCC often presents at an advanced stage, which is associated with a poorer prognosis [7]. However, early-stage OPSCC (cT1/T2N0) tends to have favourable survival outcomes [8].

There is a gradual decline in the incidence of squamous cell carcinomas in other subsites of the head and neck, however, OPSCC incidence shows a recent increase, especially in younger individuals who have lower rates of smoking and alcohol consumption [9, 10]. This increase is primarily attributed to human papilloma virus (HPV) infection, with HPV-16 being the most common etiological agent, responsible for about 90% of HPV-associated OPSCC cases [11, 12]. Males are disproportionately affected, and the most common site to be involved is the tonsil followed by the base of the tongue [13].

Early-stage OPSCC is treated using a single modality either radiation or surgery. However, definitive radiotherapy (RT) remains a major cornerstone in the management of early-stage OPSCC due to its ability to preserve organ function and achieve excellent oncological outcomes.

The need for this study arises from the growing incidence of HPV-associated OPSCC and the critical importance of optimising treatment strategies for early-stage disease. The rise in HPV-associated OPSCC highlights the changing epidemiology of head and neck cancers and shows the importance of HPV vaccination and early detection strategies to improve outcomes.

Despite the increasing prevalence, there is limited data on the long-term outcomes of definitive radiation treatment in early-stage OPSCC. Understanding the effectiveness of this treatment modality is essential for improving patient prognosis and guiding clinical decision-making. This retrospective study aims to fill this gap by assessing the treatment outcomes of early-stage OPSCC treated with definitive radiation.

## Materials and methods

The data of patients with OPSCC cT1/T2N0 treated with definitive radiation at our institution from 1st January 2015 to 31st December 2020 were retrieved from medical records. Their clinical profiles and treatment outcomes were collected using a structured proforma. Patients who were partially treated elsewhere, who received prior anti-cancer treatment and who did not report for treatment were excluded from the analysis.

The decision to include cases from 1st January 2015 to 31st December 2020 was based on the availability and completeness of the data at the time of our study’s initiation. This period allowed us to ensure a sufficient follow-up duration for assessing long-term outcomes, which is critical for the validity of our findings. Follow-up status of the included patients was updated till May 2023.

All patients were staged clinically and imaging with computed tomography (CT)/magnetic resonance imaging. None of the patients had ­positron emission tomography CT. Clinical response was evaluated initially at 3 months of treatment completion. If there was no evidence of residual disease clinically during the first follow up, is documented as a complete response. After completing the treatment, the patients were followed up every 3 months in the first year, every 4 months in the second year, and thereafter in decreasing frequency.

### Statistical analysis

Data were summarised using appropriate descriptive statistics. Locoregional relapse-free survival (LRRFS) and disease-free survival (DFS) were the primary objectives of the study. Overall survival (OS) and prognostic factors affecting treatment outcomes were the secondary objectives. LRRFS is defined as the time from the date of diagnosis till any local/regional relapse. DFS is defined as the time from diagnosis to any local/regional/systemic relapse or death due to disease. OS is defined as the time from diagnosis till death due to any cause or last follow up. Toxicity data were not assessed due to the retrospective nature of the study.

Data analysis was performed using SPSS software, version 11.0. Survival curves were obtained by using the Kaplan–Meier Method and were compared with the log-rank test. The log-rank test was used in univariate analysis to identify the potentially important prognostic variables. The variables showing statistical significance in univariate analysis were introduced step-wise into a Cox regression model to identify the independent predictors of survival. All statistical tests were two-sided, and *p* ≤ 0.05 were considered to be statistically significant.

## Results

One hundred and twelve patients with cT1/T2N0 OPSCC who were treated with definitive radiation in our centre from 1st January 2015 to 31st December 2020 were included in the analysis. The baseline characteristics are given in Table 1. The majority of the patients were males (*N* = 103, 91.9%) and 90% (*N* = 101) had habits. The median age group of the study population was 62 years. Twenty-five (22.3%) among the 112 patients had stage I disease and 87 (77.1%) had stage II disease. The most commonly affected primary site was the tonsil (*N* = 38, 33.9%) followed by the soft palate (*N* = 34, 30.3%) followed by the base of the tongue (*N* = 23, 20.5%).

After dental prophylaxis, all patients were treated with fractionated external beam RT except one patient who received brachytherapy. Ninety-seven patients (86.6%) received radiation using 2D technique, 14 (12.5%) underwent intensity modulation radiation therapy (IMRT) treatment. This distribution is primarily due to the high patient load and the limited availability of machines with IMRT capabilities. All of them completed the planned treatment; however, three patients had interruptions in between: one deferred treatment due to poor general condition, one had a 7-day gap due to grade 3 oral mucositis and one had grade 3 hyponatremia. The latter two had completed radiation without gap correction.

Hypofractionation was used in 97 patients (86.6%) where 60 Gy in 26 fractions at 2.3 Gy per fraction once daily for 5 days in a week was delivered over 5.5 weeks. These patients were treated with a mono-isocentric technique using parallel opposed lateral fields to treat the primary and upper neck nodes to a dose of 60 Gy in 26 fractions and lower anterior neck RT to a dose of 50 Gy in 20 fractions prescribed at Dmax. Fourteen (12.5%) patients received radiation using the IMRT-SIB technique to a dose of 66Gy in 30 fractions (at 2.2 Gy per fraction), 60 Gy in 30 fractions (2 Gy per fraction) and 54 Gy in 30 fractions (1.8 Gy per fraction) to high risk (CTVp1), intermediate (CTVp2) and low-risk areas (CTVn1), respectively, over 6 weeks. One patient with soft palate carcinoma was treated using high dose rate (HDR) brachytherapy delivering a dose of 48 Gy/12 fractions over 6 days 4 Gy/fraction – 2 fractions a day, minimum of 6 hours apart. None of the patients received systemic chemotherapy.

Initial assessment was done at 3 months and 119 (97.3%) patients attained remission. Three patients had residual disease and all of them had stage II OPSCC at presentation. None of them underwent salvage surgery due to inoperable recurrences. Twenty four patients (21%) relapsed and the median time to relapse was 20 months. The most frequent site of relapse was the primary site (*N* = 6, 24%) followed by regional nodes (*N* = 4, 16.6%). None of the patients had systemic relapse. Out of the 24 patients, seven (29%) underwent salvage surgery. Among the remaining 17 patients, 8 had inoperable disease, 5 were in poor general condition and 4 declined surgery. All of them received palliative treatment. Nineteen (79.1%) among the relapsed patients were of Stage II at presentation.

**Figure 1.**
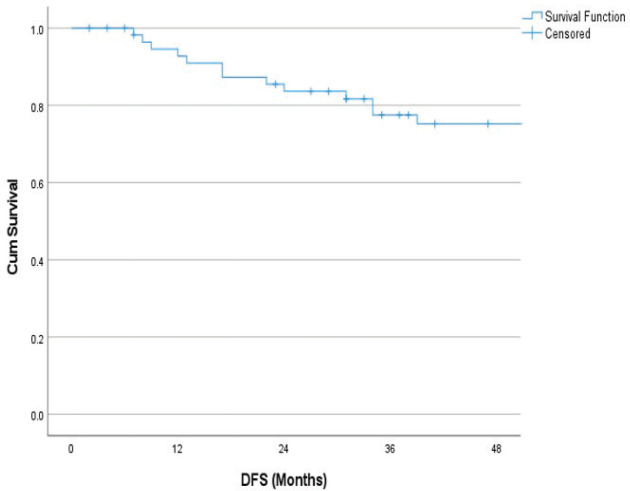
4 years loco-regional relapse free survival.

**Figure 2.**
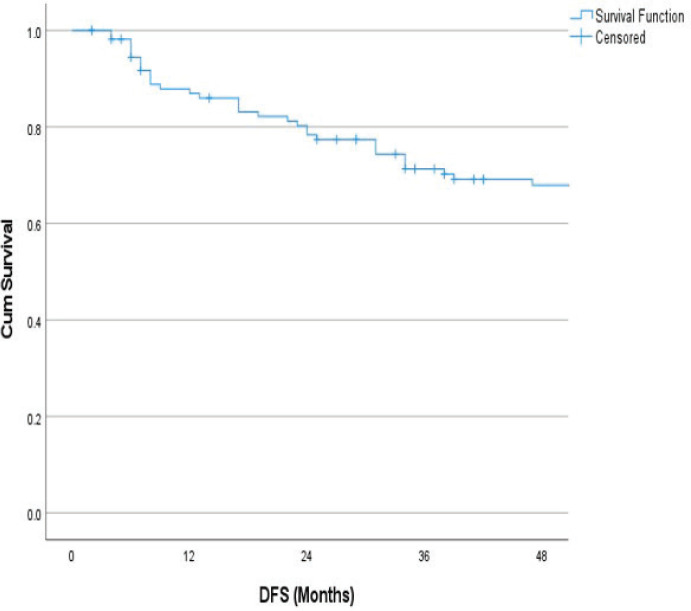
4 years DFS.

**Figure 3.**
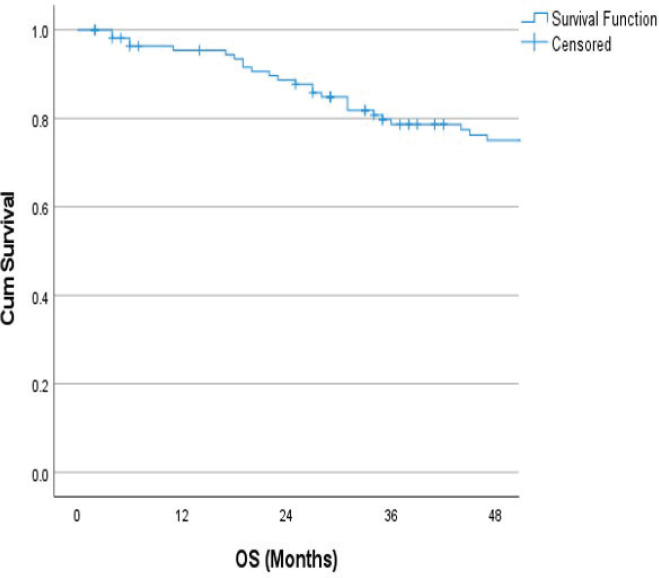
4 years OS.

**Figure 4.**
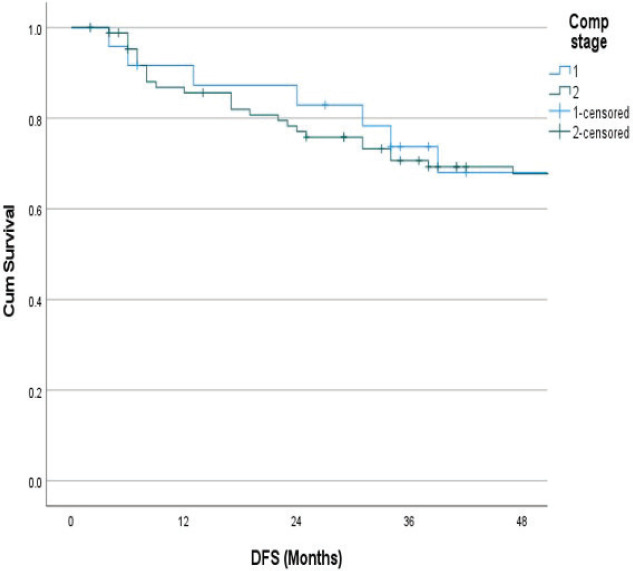
Stage wise 4 years DFS.

**Figure 5.**
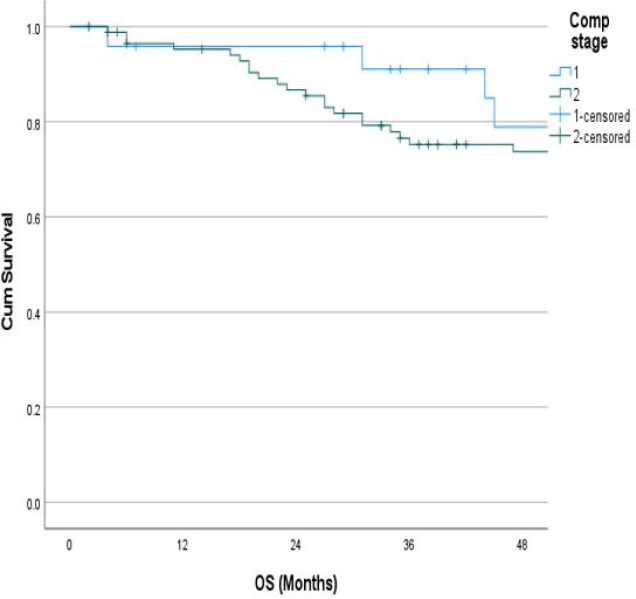
Stage wise 4 years OS.

The follow-up percentage of the data for 4 years is 77.7% and for 5 years is 69.65%. Hence, 4 years survival outcomes were calculated. The loco-regional relapse-free survival at 4 years was 75.2% (Figure 1) At a median follow up of 63 months, the 4-year DFS of the entire study cohort was 67.9% and OS was 75% (Figures 2 and 3). The stage-wise 4-year DFS and OS for stages I and II were 68.1%, 64.1%, 78.9% and 73.8%, respectively, which was not statistically significant (*p* = 0.326) (Figures 4 and 5). Also, there was no statistically significant difference in 4-year survival outcomes among various sub sites (*p* = 0.8). There was no statistically significant difference in 4-year survival outcomes between various RT schedules too (*p* = 0.1). We consolidated these various RT schedules into two groups: 60 Gy/20 fractions and others, to enhance statistical power and simplify the interpretation of results. The number of patients in other groups was too small and this approach was taken to ensure more robust and meaningful comparisons. The toxicity associated with radiation treatment was not captured in view of the retrospective nature of the study.

Five patients among the study population developed a second malignancy which was confirmed with clinical history and timing, tissue biopsy and radiological discussions. Lung was the most common site (*N* = 3, 60%). Two of them had biopsy-proven adenocarcinoma and one had squamous cell carcinoma which was radiologically more in favour of lung primary.

## Prognostic variable affecting the survival outcomes

None of the variables including gender, Eastern Cooperative Oncology Group Performance Status (ECOG PS), smoking, alcohol use, primary site, *T*-stage classification and RT dose or technique showed significance in univariate Cox regression analysis for OS and DFS (Table 2). However, age was the only significant prognostic factor that affected the treatment outcomes (*p* < 0.05). Since, only one variable showed significance in survival outcomes in univariate analysis, multivariate analysis was not done. None of the patients underwent p16 assay or HPV testing which is a significant prognostic factor for OPSCC.

## Discussion

Radiation is an effective treatment approach in early-stage oropharyngeal cancers. The 5 years local control rates reported in many series are higher than 70% to 80% [14, 15]. This demonstrates the potential of radiation in managing early-stage cancers. However, many of these studies have included patients with various stages of oropharyngeal cancers, which can skew the results. Studies reporting the outcomes of radiation in oropharyngeal cancers have only a few populations with stages I and II [14, 16, 17]. Our study specifically focuses on 112 patients with stages I and II disease and provides a more precise understanding of the effectiveness of radiation therapy in early-stage diseases.

Diagnosing at an early stage is challenging in OPSCC as they often metastasise to regional lymph nodes at presentation. This highlights the need for improved screening and diagnostic techniques to detect OPSCC earlier, potentially improving treatment outcomes.

Our study showed that 91.9% of the patients were males, which aligns with the general trend that males are more frequently affected by OPSCC. This may be attributed to lifestyle factors such as higher rates of smoking and alcohol consumption among males. Understanding these gender differences is crucial for developing targeted prevention and awareness campaigns.

The median age group of patients in our study was 62 years (range 37–82) which was similar to that reported by Cline *et al* [18] where the median age for Asians is 61.8 years. However, there is a change in the age of diagnosis of OPSCC in the Western world, with an increasing incidence of OPSCC among individuals under 45 years old. This is largely due to the association with HPV infection. This mandates the importance of HPV vaccination and public health initiatives to reduce the burden of HPV-related cancers [18–21].

The most commonly affected subsite in oropharyngeal carcinoma is the tonsils followed by the base of the tongue [9, 22, 23]. In our study, also tonsil was the most commonly affected subsite followed by the soft palate and base of the tongue.

Du﻿ring the study period, our hospital encountered significant challenges due to a high patient load and a limited number of radiation machines with IMRT capabilities. As a result, the majority of patients were treated using the 2D technique rather than the IMRT technique. This was to ensure that all patients received timely treatment. This approach, while not ideal, was essential to manage the high volume of cases and to provide the best possible care within the constraints of our resources. The reliance on 2D technique, despite its limitations compared to IMRT, shows the need for increased investment in advanced RT infrastructure to improve treatment outcomes for patients with head and neck cancers.

The 4-year OS and DFS of our study population is 75% and 67.9% which is comparable to previously reported data [7, 24–27]. Agarwal *et al* [28] reported a 3-year DFS of 80.3% and 65.8% for stages I and II OPSCC, respectively, and our study showed a 4-year DFS of 68.1% and 64.7% DFS. These findings indicate that radiation therapy is effective in maintaining disease control over a longer period.

Our study reported a locoregional control (LRC) rate of 75.2%, which aligns with the 78% LRC reported by Hicks *et al* [25] for patients treated with surgery. Parsons *et al* [29] also demonstrated similar disease control rates for OPSCC comparing surgery and RT, however, the functional outcomes were favouring RT. This suggests that radiation therapy not only provides effective disease control, but also preserves function, which is very important for patients’ quality of life. The OS and LRC of stages I/II OPSCC from various studies are summarised in Table 3.

Despite the better local control rate, locoregional failure is the most common form of disease recurrence. In our study, 24 patients relapsed at a median follow up of 63 months, of which the majority was in the primary site (*N* = 20, 83%) followed by regional nodes (*N* = 4, 17%). None of the patients developed distant metastasis. Nineteen of the 24 patients were of stage II disease. Tonsil was the most common primary site to be relapsed among other subsites (*N* = 10, 41.6%). Garden *et al* [30] in a series of 776 OPSCC patients demonstrated a primary site recurrence of 7% and nodal recurrence of 3%, following radiation, which is much less compared to our study. This highlights the importance of vigilant follow-up and monitoring for local recurrences, especially at the primary site.

The salvage rates in our study were poor, with only 7 out of 24 undergoing salvage surgery and this shows the challenges in managing recurrent OPSCC. Several factors contributed to this outcome. First, many patients presented with inoperable recurrences, making surgical intervention unfeasible. Second, the poor general condition of some patients precluded them from undergoing major surgical procedures due to the high risk of complications. Additionally, a number of patients were unwilling to undergo salvage surgery, mainly due to concerns about the potential risks and impact on their quality of life. These emphasise the need for careful patient selection and consideration of individual circumstances when planning salvage treatment.

The majority of recurrences occurred within the first 2 years after treatment completion which underscores the need for a close follow up during this period. Early detection of recurrences can lead to better salvage therapy outcomes and improved disease control. In a series reported by Röösli *et al* [31] out of 153 recurrences, only 51 (12%) could be salvaged. Choi *et al* [32] demonstrated salvage surgery in more than 50% of the relapsed cases, with a 2-year OS of 56.4%. In another series by Omura *et al* [33], 18 out of 23 patients with recurrent OPSCC following radical RT were salvaged using surgery. Therefore, salvage treatment in recurrent OPSCC is feasible; however, the patients should be selected very carefully.

The incidence of second malignancy was only 4.4% and lung was the most common site. This warrants the importance of vigilant monitoring and implementing preventive measures like smoking cessation programs, which can further reduce the risk of developing secondary lung cancers and improve overall patient outcomes.

Ryan Camilon *et al* [34] reported that survival outcomes worsen with increasing age, particularly after 65 years. Similarly, in our study, age was the only statistically significant prognostic factor affecting treatment outcomes. Patients above 60 years of age had a negative impact on survival, likely due to the comorbidities and effects of aging which make them susceptible to the pathogenesis of OPSCC. This shows the need for tailored treatment approaches and comprehensive management of comorbid conditions in older patients to improve their survival outcomes.

None of the patients underwent p16 assay or HPV testing which is a significant prognostic factor for OPSCC. Although the AJCC incorporated HPV testing into the staging guidelines in 2018, our hospital only introduced routine testing in late 2020. This delay was primarily due to the COVID-19

crisis, which significantly impacted our resources and priorities. As a result, we were unable to include HPV status in our analysis, which may affect the interpretation of our findings. Generally, p16-positive patients present with node-positive disease; however, our study group has only T1/T2 diseases.

Our study has several limitations that should be acknowledged. First, its retrospective nature may introduce biases and limit the ability to establish causality. Second, the non-availability of HPV status is an important limitation, as HPV is a known risk factor for OPSCC and can influence treatment outcomes. Third, the relatively small sample size reduces the statistical power and generalisability of the findings. A larger cohort could enhance the robustness and generalisability of our results.

One of the significant limitations of our study was the inability to capture data on the toxicity associated with radiation treatment, especially with a dose of 2.3 Gy per fraction using the 2D technique. Although we initially aimed to collect detailed information on treatment-related toxicities, the retrospective nature of the study posed considerable challenges. The variability in documentation practices and the incomplete records from the study period hindered our ability to gather a complete and consistent dataset on toxicity outcomes. This is a major limitation, as it may impact the ability to fully assess the safety profile of the treatment regimen used.

Additionally, the majority of patients in our study received radiation using the 2D technique. It is well-established that IMRT is associated with lower toxicity compared to 2D technique. However, due to the retrospective nature of the study, we were unable to systematically document or compare the side effects of each technique.

Despite these limitations, the study provides valuable insights based on the available complete and reliable data within the specified timeframe. Future prospective studies with standardised data collection methods are needed to better evaluate the toxicity and overall efficacy of higher doses per fraction of radiation in this patient cohort.

Additionally, research with a prospective design, inclusion of HPV status and a larger sample size would help to validate and expand upon these findings.

## Conclusion

The survival outcomes of early-stage OPSCC following definitive radiation therapy are consistent with those reported in the literature, demonstrating the effectiveness of this treatment approach. Age emerged as the only significant prognostic factor, with older patients experiencing poorer survival outcomes, likely due to comorbidities and the effects of aging. Loco-regional recurrence remains the most common form of disease recurrence, and the salvage rates are notably poor. This highlights the need for close monitoring, especially within the first 2 years post-treatment, and the importance of personalised treatment plans to improve patient outcomes.

## Conflicts of interest

All authors declare that we have no significant competing interests that might have influenced the work described in the manuscript.

## Funding

Not applicable.

## Patient consent for publication

Not applicable being a retrospective analysis.

## Ethics

The expedited review committee - The Human Ethics Committee Regional Cancer Centre Trivandrum, examined, discussed and approved the study. HEC No: 09/23.

## Availability of data and materials

Data were retrieved using case records of patients and compiled together for analysis.

## Author contributions

ZS performed the literature search, collected the data, wrote the manuscript, prepared the tables and edited the manuscript. RRK helped in the design of the study, helped in data collection and analysis. MR and LMN helped in the data collection, preparation of tables and editing manuscript. NJ and JMM helped in updating follow up information and writing manuscript. KP and IS helped in analysis of data and writing up discussion. JNK helped with statistical analysis and preparation of figures and tables. KCT performed literature searches, designed the study and also helped in writing and editing the manuscript. All authors read and approved the final version of the manuscript.

ZS- Zuzaki Shabin

RK- Rejnish Kumar

MR- Malu Rafi

LMN- Lekha M Nair

KP – Krishnapriya P

NJ- Nijo Jose

JMM- John M Mathew

IS- Indu Sasikumar

JNK- Jagathnath Krishna K M

KCT – Kainickal Cessal Thommachan.

## Figures and Tables

**Table 1.** Baseline characteristics of the study population.

Patient characteristics	*N* = 112 (%)
Age group	
≤ 60 years	62 (55.4)
>60 years	50 (44.6)
ECOG PS	
1	109 (97.4)
2	3 (2.6)
Gender	
Females	9 (8.03)
Males	103 (91.97%)
Habits	
Smoking	100 (89.2)
Alcohol	90 (80.35)
Chewing	32 (28.5)
Comorbidities	
No	86 (76.7)
Yes	26 (23.3)
Primary site	
Tonsil	38 (33.9)
Base tongue	23 (20.5)
Soft palate	34 (30.3)
Uvula	8 (7.2)
Posterior pharyngeal wall	5 (4.5)
Vallecula	4 (3.6)
T stage	
T1	26 (23.3)
T2	86 (76.7)
Composite stage	
I	26 (23.3)
II	86 (76.7)
RT dose	
60 Gy/26 fr	90 (80.4)
55 Gy/20 fr	7 (6.2)
66 Gy/30 fr	14 (12.5)
48 Gy/12 fr	1 (0.9)
Technique	
2D	97 (86.6)
IMRT	14 (12.5)
HDR brachytherapy	1 (0.9)

**Table 2.** 4 years OS and DFS by variables.

Variable	4 years OS (*p* value)	4 years DFS (*p* value)
Age		
< 60 years	85.1 (<0.001)	76.6 (*p* = 0.026)
>60 years	65.1	60.6
Gender		
Female	87.5 (*p* = 0.533)	62.2 (*p* = 0.964)
Male	73.8	68.2
Primary site		
Tonsil and pillars	71.9 (*p* = 0.775)	66.9 (*p* = 0.814)
Base tongue	63.9	56.6
Soft palate	86.7	80.5
Uvula	83.3	72.9
Posterior pharyngeal wall	60.0	60.0
Vallecula	75.0	50.0
Composite stage		
I	78.9 (*p* = 0.326)	68.1(*p* = 0.723)
II	73.7	67.8
RT dose		
60 Gy/26 fr	75.1 (*p* = 0.161)	68.3 (*p* = 0.314)
Others	74.7	67.8

**Table 3.** OS and LRC of stages I/II OPSCC from various studies.

Study (year)	Number of patients	OS(%)	LRC(%)	Treatment
Current study (2023)	112	75% (4 years)	75.2% (4 years)	Radical radiation
Pedro *et al* (2017) [24]	42 (S) versus 19 (RT)	75 versus 81 (5 years)	49 versus 72 (5 years)	Surgery +/- RT versus RT +/- Chemo
Valakh *et al* (2014) [26]	386 (S) 362 (RT)	66.1 (S) 50.2 (RT) (5 years)	-	Surgery versus radiation
Selek *et al* (2003) [7]	175	70% (5 years)	81% (5 years)	Radical radiation
Hicks *et al* (1991) [25]	20 (S) versus 4 (RT)	61% (Surgery) 37% (RT) (5 years)	78% (Surgery) 75% (RT) (5 years)	Surgery versus radiation
Lammare *et al* (2011) [27]	17 (S) versus 26 (RT)	93% versus 72% (5 years)	69 versus 89 (5 years)	Surgery versus radiation
